# Membrane Pore Formation Unveiled by ∞RETIS Path Sampling: From Thinning to Flip-Flop

**DOI:** 10.1021/acs.jctc.5c01814

**Published:** 2026-01-17

**Authors:** Daniel Tianhou Zhang, Lukas Baldauf, Grzegorz Lazarski, Titus S. van Erp, Wataru Shinoda

**Affiliations:** † Research Institute for Interdisciplinary Science, 12997 Okayama University, 3-1-1 Tsushima-naka, Okayama 700-8530, Japan; ‡ Department of Chemistry and Biomedical Science, 8018 Norwegian University of Science and Technology, Trondheim 7491, Norway

## Abstract

Pore formation in lipid bilayers plays a vital role in membrane fusion, transport, and signaling. Yet, its detailed mechanism remains elusive due to the limitations of conventional simulation methods. To overcome this, we apply a newly developed path sampling technique, the asynchronous and infinite swap version of Replica Exchange Transition Interface Sampling (∞RETIS), to study pore formation in a dimyristoylphosphatidylcholine (DMPC) bilayer modeled with the CHARMM36m force field. Our results reveal a sequence of tightly coupled events: pore nucleation sites are determined by early-stage thinning, and the progress into a metastable pore requires a combination of polar defects and close proximity between lipids across opposite leaflets. Using *Inf-init*, an initiation protocol based on ∞RETIS, rare trajectories can be generated starting directly from equilibrium simulations. *Inf-init* and ∞RETIS simulations reveal that lipid flip-flop occurs exclusively via local membrane thinning, and pore closure often results in asymmetric lipid distributions.

## Graphical abstract

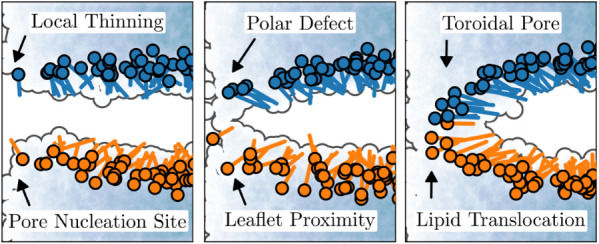

## 1 Introduction

Pore formation in lipid bilayers is a fundamental process underlying biologically significant phenomena, including regulated cell death, membrane permeability, solute leakage, and lipid flip-flop.^1–5^ Despite its importance, elucidating the molecular mechanism of pore formation remains challenging due to the transient and highly dynamic nature of its intermediate and transition states. Direct experimental characterization of such fleeting events is notoriously difficult, which has motivated the use of molecular dynamics (MD) simulations to obtain atomistic insights. However, conventional MD is fundamentally limited by the disparity between the femtosecond time step and the millisecond time scales on which many rare events occur. As a result, brute-force simulations become prohibitively inefficient for sampling barrier-crossing events, and the mechanistic picture of pore formation has remained incomplete.

Enhanced sampling methods such as umbrella sampling (US) can alleviate these difficulties by biasing the dynamics along predefined collective variables (CVs).^6^ While powerful for estimating thermodynamic profiles, these techniques distort dynamical properties and critically depend on the choice of an appropriate CV. In the context of pore formation, CVs have indeed been refined over time to capture polar defects and pore expansion.^7–12^ Still, even with these advances, biased approaches cannot provide an unbiased statistical description of the transition pathways.

Path sampling methods offer a fundamentally different solution. Instead of biasing the dynamics, Transition Path Sampling (TPS) and Transition Interface Sampling (TIS) generate statistically rigorous ensembles of unbiased trajectories that connect stable states.^13–15^ This trajectory-based framework avoids reliance on a perfect reaction coordinate, thereby uncovering mechanistic pathways without imposing external constraints. In essence, path sampling directly targets the rare reactive trajectories that conventional simulations fail to observe, providing both kinetic and thermodynamic information with minimal assumptions.

Recent algorithmic developments have substantially expanded the scope of path sampling methods. In particular, Replica Exchange TIS (RETIS) introduced efficient path swaps to enhance sampling efficiency.^16^ However, due to the incorporation of RE moves, RETIS is most efficiently implemented as a largely sequential algorithm, which may limit its applicability on modern parallel hardware.^17,18^ Considerable effort has therefore been devoted to parallelizing RETIS, leading to the development of the ∞RETIS method. ∞RETIS enables parallel and asynchronous generation of rare MD trajectories while simultaneously performing replica exchange across ensembles at the infinite-swap limit.^17,18^ By decoupling path generation from ensemble coordination and exploiting parallel computing, this innovation achieves dramatic acceleration, up to tens of times faster than standard RETIS and potentially even greater when abundant computational resources are available. With these methodological breakthroughs, processes that were once far beyond the reach of unbiased trajectory-based simulations have become tractable.

In this work, we demonstrate how ∞RETIS can be applied to lipid bilayer pore formation, a prototypical example of a biologically meaningful, rare, and mechanistically complex event. By harnessing the efficiency of ∞RETIS, we can resolve the sequence of molecular steps underlying pore nucleation, metastable pore stabilization, lipid flip-flop, and pore closure. Beyond the specific insights into DMPC bilayers, our study illustrates how modern path sampling techniques open new avenues for exploring rare membrane phenomena and, more generally, for expanding the scientific frontier of unbiased simulations of complex biomolecular processes.

## 2 Methodology

### 2.1 A Summary of ∞RETIS Theory

∞RETIS is a replica exchange Monte Carlo based method for calculating kinetics by sampling unbiased, rare MD trajectories between stable states separated by energy barrier(s). While multistate RETIS variants exist^19,20^ we focus here on the original two-state (A and B) formulation. ∞RETIS relies on a one-dimensional CV, denoted the order parameter, λ, which characterizes the transition progress along a trajectory. Two terminal interfaces λ_*A*_ < λ_*B*_ define the boundaries of the stable states: a phase point *x* lies within state A if λ­(*x*) < λ_*A*_ and within state B if λ­(*x*) > λ_*B*_. By defining a set of nonoverlapping interfaces {λ_0_, λ_1_, λ_2_, ···, λ_*n*_} with λ_0_ = λ_*A*_ and λ_*n*_ = λ_*B*_, we obtain the path ensembles [0^–^], [0^+^], ···, [(*n*–1)^+^]. The [0^–^] ensemble samples trajectories that explore the interior of state A, with both end points lying above λ_*A*_. The [*i* ^+^] ensembles sample paths originating in A, crosses λ_*i*_, and terminates either in A or in B. The rarity and the average length of the sampled path distributions increases with the path ensemble [*i* ^+^], with primarily the highest path ensembles sampling reactive trajectories.

The sampled trajectories in an [*i* ^+^] ensemble can be utilized to compute the conditional probability that a trajectory also crosses the next interface λ*_i_*_+1_ given that it has crossed λ_*i*_, denoted as *P*_*A*_(λ*_i_*_+1_|λ*_i_*). The interfaces are positioned to ensure suitable crossing probabilities between successive levels, typically *P*_*A*_(λ*_i_*_+1_|λ*_i_*) ≈ 0.2–0.4, enabling accurate estimation of the transition rate constant *k*_*AB*_ via

1 $$k_{AB}=f_{A}P_{A}\left(\lambda_{B}|\lambda_{A}\right)=f_{A}\overset{n-1}{\underset{i=0}{\prod }}P_{A}\left(\lambda_{i+1}|\lambda_{i}\right)$$

The flux *f*_*A*_ is calculated from the average path lengths of the [0^–^] and [0^+^] ensembles, and the individual crossing probabilities *P*_*A*_(λ*_i_*_+1_|λ*_i_*) are obtained from the corresponding [*i* ^+^] ensembles. While both the flux *f*_*A*_ and the conditional crossing probability *P*_*A*_(λ*_B_* |λ_*A*_) depend on the placement of the λ_*A*_ interface, their product yield the rate constant, which is independent of this choice.

New trajectories are generated using *shooting moves*: a phase point along an existing path is selected, its velocities are perturbed, and the system is propagated forward and backward in time until either state A or B is reached. Acceptance or rejection follows a Metropolis criterion, ensuring detailed balance. More efficient shooting-based moves like the wire-fencing (WF) move, in combination with high-acceptance ensembles, have also been developed.^21,22^ For the rest of the text, the term *shooting move* refers to both standard and WF variants. In addition to shooting moves, replica exchange moves are performed to sample paths in other ensembles. To maximize swapping efficiency, an (in principle) infinite number of swaps are assumed between the [*i* ^+^] ensembles.^23^ To swap paths between the [0^–^] and [0^+^] ensembles, the point-exchange move is performed.^16^ ∞RETIS also allows multiple shooting moves to run asynchronously in parallel, accelerating path generation when more computational resources are available. Details of the ∞RETIS simulations are provided in Section II D.

### 2.2 Collective Variables

To apply ∞RETIS for the study of pore formation in lipid bilayers, several candidate order parameters λ were explored. Among these are the well-established pore formation and expansion CVs ξ_ch_ and ξ_p_ that have been shown to effectively describe pore formation in umbrella sampling studies.^10,11^ As discussed in Section III, additional CVs were required to capture other structural features that arose concurrently with pore formation during the ∞RETIS simulations, like lipid translocation events. Below, we briefly summarize the CVs used in the ∞RETIS simulations and subsequent analysis. The CVs are also shown in pore formation and lipid translocation trajectories (Movie S1 and Movie S2).

#### 2.2.1 Pore Formation and Expansion (ξ_ch_, ξ_p_)

Pore formation in lipid bilayers is characterized by the chain coordinate ξ_ch_,^10^ which quantifies the formation of a continuous polar defect across the membrane by counting the presence of polar heavy atoms (oxygen atoms in water molecules or lipid phosphate groups) within a membrane-spanning cylindrical volume aligned along the bilayer normal *z*. The cylinder is divided into discrete slices along *z*, and the ξ_ch_ is defined as the fraction of slices containing polar heavy atoms. An individual slice is defined to be fractionally occupied if the slice contains one atom (typically 0.75), and completely filled (≈1) if the slice contains two or more atoms. ξ_ch_ ranges from 0 to 1, and yields ξ_ch_ = 0.75 if each slice occupies a single atom. The term “continuous polar defect” is therefore defined here for when all the slices are at least partially occupied (ξ_ch_ > 0.75). Figure 1A illustrates ξ_ch_, showing a 26-slice cylinder with a radius of 0.8 nm and length 2.6 nm (0.1 nm × 26), corresponding to the default ξ_ch_ parameters used for dimyristoylphosphatidylcholine (DMPC) bilayers. In the same DMPC system, a defect-free state corresponds to ξ_ch_ ≈ 0.2–0.4, while a metastable open pore appears beyond the transition state at ξ_ch_ ≈ 0.88 (CHARMM36m force field, 300 K; see Figure 3B). The pore-expansion coordinate ξ_p_ was introduced to capture subsequent pore growth by incorporating the number of polar atoms within the pore, and may take values larger than 1.^11^ We used the default DMPC parameters for ξ_ch_ and ξ_P_. For the palmitoyloleoylphosphatidylcholine (POPC) bilayer, the number of cylinder slices was increased to 30.

**Figure 1:**
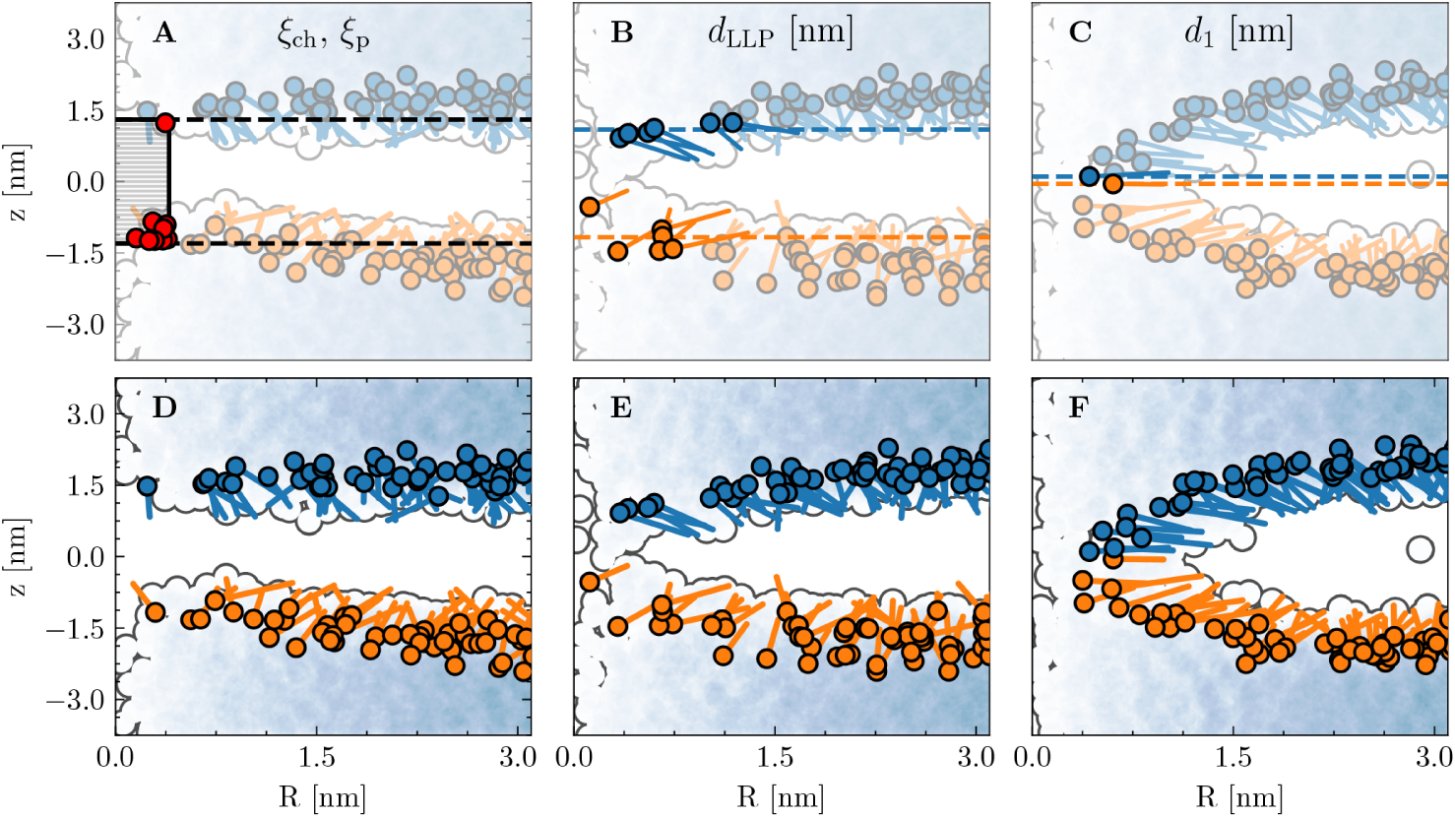
A membrane normal (*z*) and radial (*R*) representation of three geometries from the predicted ξ_ch_ cylinder *xy*-center, shown in panels A–C, and again in panels D–F, without CV visualizations. The lipid head–tail representation is based on the lipid phosphorus atom and center of mass, with colors assigned according to the *d*_1_ grouping scheme. The oxygen atoms of water molecules are shown as a blue-white density scatter plot. Panel A: the ξ_ch_ cylinder is depicted along with the enclosed heavy polar atoms in red. The black dashed line indicates the additional region covered by ξ_p_. Panel B: the selected *N*_LLP_ = 12 lipids and the mean *z* positions of the two sets are shown. Panel C: the two selected *d*_1_ lipids and their phosphate *z* positions are highlighted in blue and orange. These geometries correspond to Figure 4G1,G4 and G7.

For clarity, we briefly reiterate how the cylinder is positioned in the *xy* plane of the bilayer. During ξ_ch_ calculation, polar heavy atoms within the cylinder are grouped into layers spanning the full *xy* plane. The *xy* coordinates of the cylinder are then set to the average of the centers of masses of each layer, ensuring that the position is not biased by atoms in the outermost layers. The center-of-mass computation is well-defined under periodic boundary conditions.^24^ Figure 2A–B illustrates the resulting *xy* localization for pore-free and open-pore geometries.

**Figure 2:**
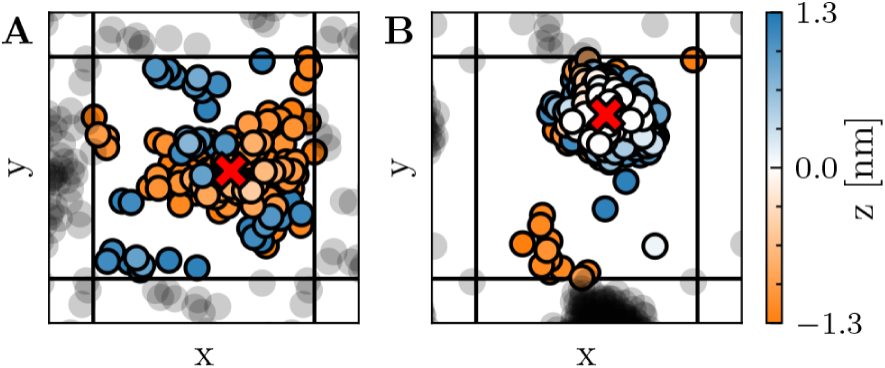
The position of the ξ_ch_ cylinder in the *xy* plane is indicated by a red cross for two geometries, A and B (corresponding to Figure 4G1,G7), with ξ_ch_ = 0.36 and ξ_ch_ = 1.0, respectively. The circles represent polar heavy atoms located within 1.3 nm of the membrane center. The plotting order of these atoms is determined by their proximity to the membrane center along the *z*-axis. Only the visualized atoms within the 0.8 nm cylinder radius are included in the ξ_ch_ calculation. Periodic images and atoms are shown in black.

#### 2.2.2 Local Leaflet Proximity (*d*_LLP_)

We define a local leaflet proximity CV, *d*_LLP_, that measures the vertical (*z*) separation between opposing leaflets by considering phosphate atoms in the lipids,

2 $$d_{\mathrm{LLP}}={\mathrm{z̅}}^{U}-{\mathrm{z̅}}^{L}$$

*d*_LLP_ is evaluated within a specified local *xy* region, here chosen as the dynamically positioned ξ_ch_ cylinder. The procedure is

(1) Select the *N*_LLP_ lipids closest to the ξ_ch_ cylinder center in the *xy* plane.
(2) Sort them into upper {*U*} and lower {*L*} sets based on their *z* coordinates relative to the membrane center of mass.
(3) If one set is empty, include additional lipids until both contain at least one.
(4) Compute the mean *z* positions of each set (*z̅*) and take their difference to obtain *d*_LLP_.

Figure 1B illustrates *N*_LLP_ = 12, the value used in all calculations.

#### Lipid Translocation (*d*_1_, *d*_2_, $d_{1}^{*}$ , $d_{2}^{*}$ , *d*_CNT_)

2.2.3

To capture lipid translocation between leaflets without explicit lipid tracking, we define

$$d_{1}=z_{1}^{U^{\prime }}-z_{1}^{L^{\prime }}$$ 3

where *d*_1_ is the *z*-axis distance between the lipid pair from the upper and lower lipid set {*U*′} and {*L*′} that yield the lowest Euclidean distance. For bilayers with an even number of lipids, {*U*′} and {*L*′} each contain half the total, sorted by the *z* coordinates of their phosphorus atoms. *d*_1_ is positive and, in a symmetric bilayer *z*(−0/+0), close to but slightly smaller than the average membrane thickness. A small *d*_1_ thus indicates that the selected lipid pair is near the bilayer center (Figure 4G6) or that a translocation event has occurred (Figure 4G8).

In a defect-free bilayer (ξ_ch_ ≈ 0.2), *d*_1_ ≈ 0 implies an imbalance (e.g., −1/+1) but cannot distinguish larger asymmetries. A coarse distinction between −0/+0, −1/+1, and −2/+2 states can be made by also considering the second-closest pair, *d*_2_,

$$d_{2}=z_{2}^{U^{\prime }}-z_{2}^{L^{\prime }}$$ 4

excluding the lipids used in *d*_1_. The sum *d*_1_ + *d*_2_ thus differentiate 0, 1, and 2 translocated lipids (Figure 9B–C).

The ∞RETIS simulations reported in Section II D employed the earlier variants $d_{1}^{*}$ and $d_{2}^{*}$ , in which lipid pairs were selected based solely on minimal separation along the *z* direction rather than Euclidean proximity. These variables spuriously decrease during large membrane undulations; we therefore recommend the use of *d*_1_ and *d*_2_ for future studies.

Finally, we introduce *d*_CNT_, a CV that quantifies leaflet asymmetry as the difference in the number of lipids in the upper and lower leaflets:

$$d_{\mathrm{CNT}}=\left|\frac{N_{lip}}{2}-\overset{N_{lip}}{\underset{i=1}{\sum }}1\left(z_{i}>z_{COM}\right)\right|$$ 5

where *N*_lip_ is the total number of lipids in the system and **1** is an indicator function that equals 1 if *z*_*i*_ > *z*_COM_ and 0 otherwise. Here, *z*_*i*_ denote the *z*-coordinate of the phosphorus atom of lipid *i*, and *z*_COM_ is the *z*-coordinate of the center of mass of the lipid bilayer. For bilayers with an even number of lipids, *d*_CNT_ = 0, 1, 2 correspond to leaflet imbalances of −0/+0, −1/+1 and −2/+2, respectively.

### 2.3 Molecular Dynamics Setup

The main MD system for ∞RETS simulations consisted of 128 DMPC lipids and 5,749 water molecules. The initial configuration was prepared using CHARMM-GUI and simulated with GROMACS 2024.5 employing the CHARMM36m force field and TIP3P water.^25–27^ The velocity Verlet integrator was used with a 2 fs time step. Temperature and pressure were maintained at 300 K and 1 bar using the V-rescale thermostat and the C-rescale barostat.^28,29^ Two additional bilayers were simulated; (i) 256 DMPC + 11,452 waters, and (ii) 256 POPC + 11,511 waters, both with 29 K^+^ and 29 Cl^–^ ions. Both used identical MD parameters.

Simulations were primarily performed on nodes equipped with two NVIDIA GeForce RTX 4080 SUPER GPUs and a single Intel Xeon w5-2565X CPU, providing a throughput of approximately 1,700 ns/day for eight 128-DMPC replicas or 980 ns/day for the 256-lipid systems.

### 2.4 ∞RETIS Simulations

To study pore formation (PF) and pore closing (PC), four ∞RETIS simulations were performed, each defined by its main order parameter λ.

- **PF**_**0**_: Used ξ_ch_ with terminal interfaces λ_*A*_ = 0.4 and λ_*B*_ = 0.99.
- **PF**_**1**_: Pore formation and lipid translocation starting from a defect-free bilayer (*d*_CNT_ = 0).
- **PF**_**2**_: Pore formation and translocation starting from any asymmetric bilayer (*d*_CNT_ ∈ {1, 2, 3, ···}).
- **PC**_**1**_: Pore closing into any defect-free bilayer (*d*_CNT_ ∈ {0, 1, 2, 3, ···}).

PF_1_ and PF_2_ used a conditional (*if-else*) order parameter λ based on ξ_p_, $d_{1}^{*}$ and $d_{2}^{*}$ , whereas PC_1_ employed only ξ_p_. Interface placements for PF_1_, PF_2_ and PC_1_ are shown in Figure 9A–C.

For all simulations PF_1_, PF_2_, and PC_1_, the [0^–^] and [0^+^] ensembles used standard shooting moves, while the [*i* ^+^] ensembles used the wire-fencing move with a subtrajectory number of 3 and high-acceptance ensembles, following prior ∞RETIS work.^18,30^ Eight workers were employed with 22 path ensembles for PF_0_, 25 for PF_1_–PF_2_, and 17 for PC_1_. Subcycle numbers were 1,000 (PF_0_, PC_1_) and 5,000 (PF_1_, PF_2_), but all trajectories were saved at a 1000 subcycle resolution. When interface placement proved suboptimal, simulations were restarted with optimized intermediate interfaces, while keeping λ_*A*_ and λ_*B*_ fixed. When multiple compute nodes were available, PF_1_, PF_2_ and PC_1_ runs were executed in parallel. Data in Figure 9 and Table 2 combine results from four, three, and two independent PF_1_, PF_2_, and PC_1_ simulations, respectively. All ∞RETIS simulations were performed using version 2025.4 of the open-source Python implementation *infretis* (github.com/infretis/infretis). Example input files are available at github.com/infretis/infentory. *Inf-init* and Data analysis was performed using inftools (github.com/infretis/inftools), which relies in part on NumPy^31^ and MDAnalysis.^32^

## 3 Results and Discussion

### 3.1 Local Membrane Thinning and the Transition State

We initially performed an ∞RETIS simulation (PF_0_) across the collective variable ξ_ch_ for a 128-DMPC lipid bilayer system using the CHARMM36m force field at 300 K, as described by Hub and Awasthi.^10^ From the sampled trajectories generated by the ∞RETIS simulation, spanning the initial and final interfaces at λ_*A*_ = 0.40 and λ_*B*_ = 0.99, we observed that trajectories approaching a maximum ξ_ch_ value of ≈0.99 frequently returned to λ_*A*_, even after crossing the proposed transition state at ξ_ch_ ≈ 0.88 identified via umbrella sampling. This behavior is visualized by the computed conditional crossing probability *P*(λ|λ_*A*_) that does not display the characteristic “point-of-no-return” plateau in the vicinity of the predicted US transition state (TS), shown in Figure 3A. Rather, as ξ_ch_ increases from 0.88 to 0.99, *P*(λ|λ_*A*_) decreases by an order of magnitude (from 5.20 × 10^–9^ to 3.82 × 10^–10^). These findings suggest that even reactive trajectories starting in λ_*A*_ and reaching beyond λ_*B*_ may quickly return to the initial state if the MD simulations are continued. This implies that the formation of a continuous polar defect through the membrane (ξ_ch_ ≈ 0.9–1.0) alone may be insufficient to achieve or characterize a metastable pore state.

**Figure 3:**
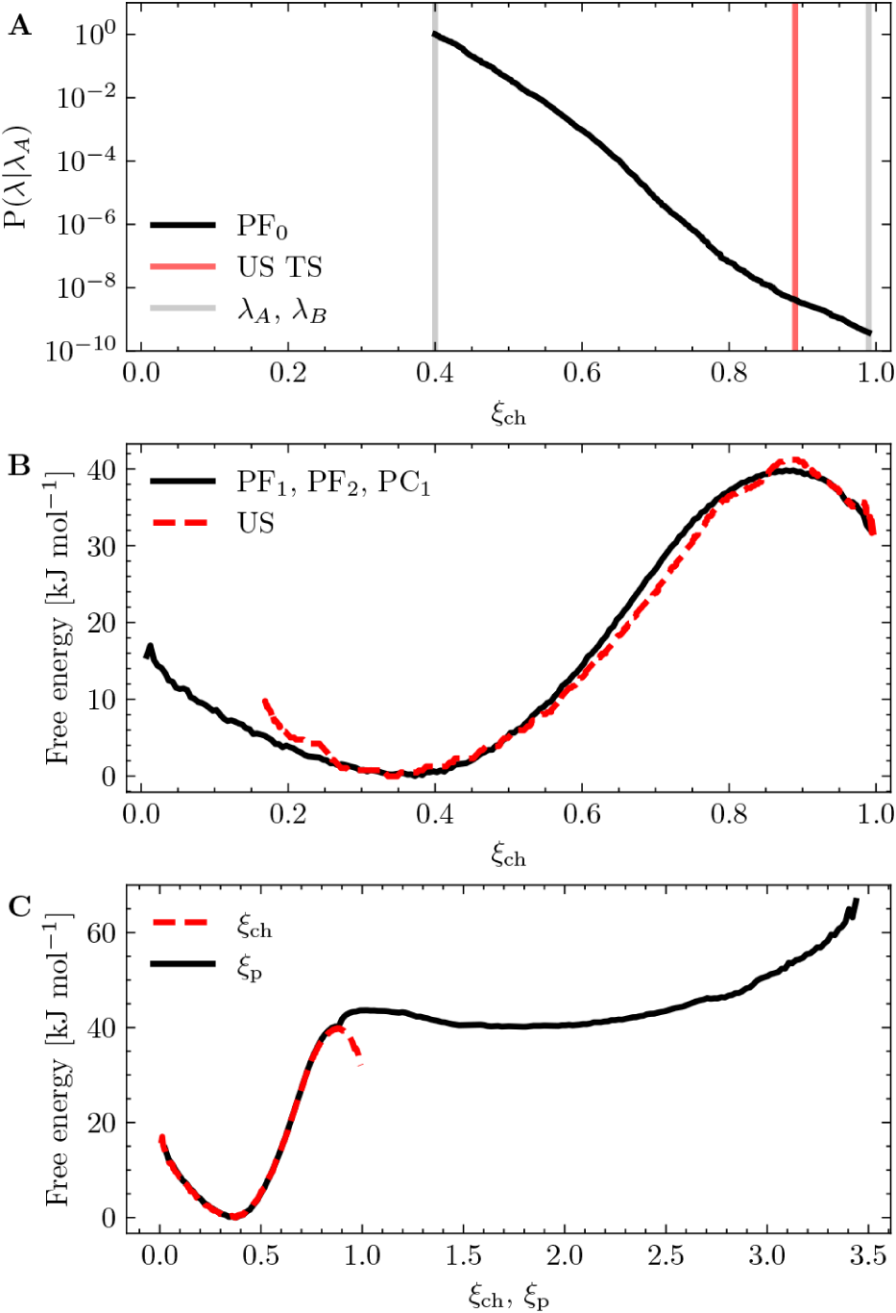
Panel A: The conditional crossing probability as a function of ξ_ch_, computed from an ∞RETIS simulation with terminal interfaces at λ_*A*_ = 0.4 and λ_*B*_ = 0.99. The predicted US TS is shown in red. Panel B: A comparison between the original ξ_ch_ publication (reproduced from Ref 10. Copyright 2017 American Chemical Society) and the combined PF_1_, PF_2_ and PC_1_ results. The projected free energy across ξ_ch_ is obtained by matching the forward and backward conditional energies of PF_1_ and PF_2_ with PC_1_.^33^ Panel C: A comparison between the projected PF_1_, PF_2_ and PC_1_ free energy onto ξ_ch_ and ξ_p_.

To investigate this inconsistency between umbrella sampling and the results from ∞RETIS simulation PF_0_, we visualized trajectory frames reaching high ξ_ch_ values and qualitatively categorized pore-opening events into several general types: (1) polar defects occurring without lipid headgroups near the membrane center (Figure 4G3), (2) polar defects where headgroups from one leaflet approach the center (Figure 4G4), and (3) polar defects involving headgroups from both leaflets in proximity to the membrane center (Figure 4G5). These snapshots, all having polar defects of ξ_ch_ ≈ 0.85, provide insights into the diversity of polar defect structures that may form in bilayers. The geometries suggest that the stabilization of a metastable pore likely requires the translocation of multiple lipids from both leaflets toward the membrane center in addition to the formation of a continuous polar defect (ξ_ch_ > 0.9). Therefore, relying solely on a polar defect definition that does not differentiate between polar heavy atoms belonging to water or lipids may be insufficient to fully describe the metastable open pore, a requirement for path-sampling simulations that depend on well-defined stable states. We note here, however, that pore formation was not guaranteed even when a trajectory crossed ξ_ch_ ≥ 0.88 with one or two lipid head-groups from a single leaflet crossing the center of the membrane.

**Figure 4:**
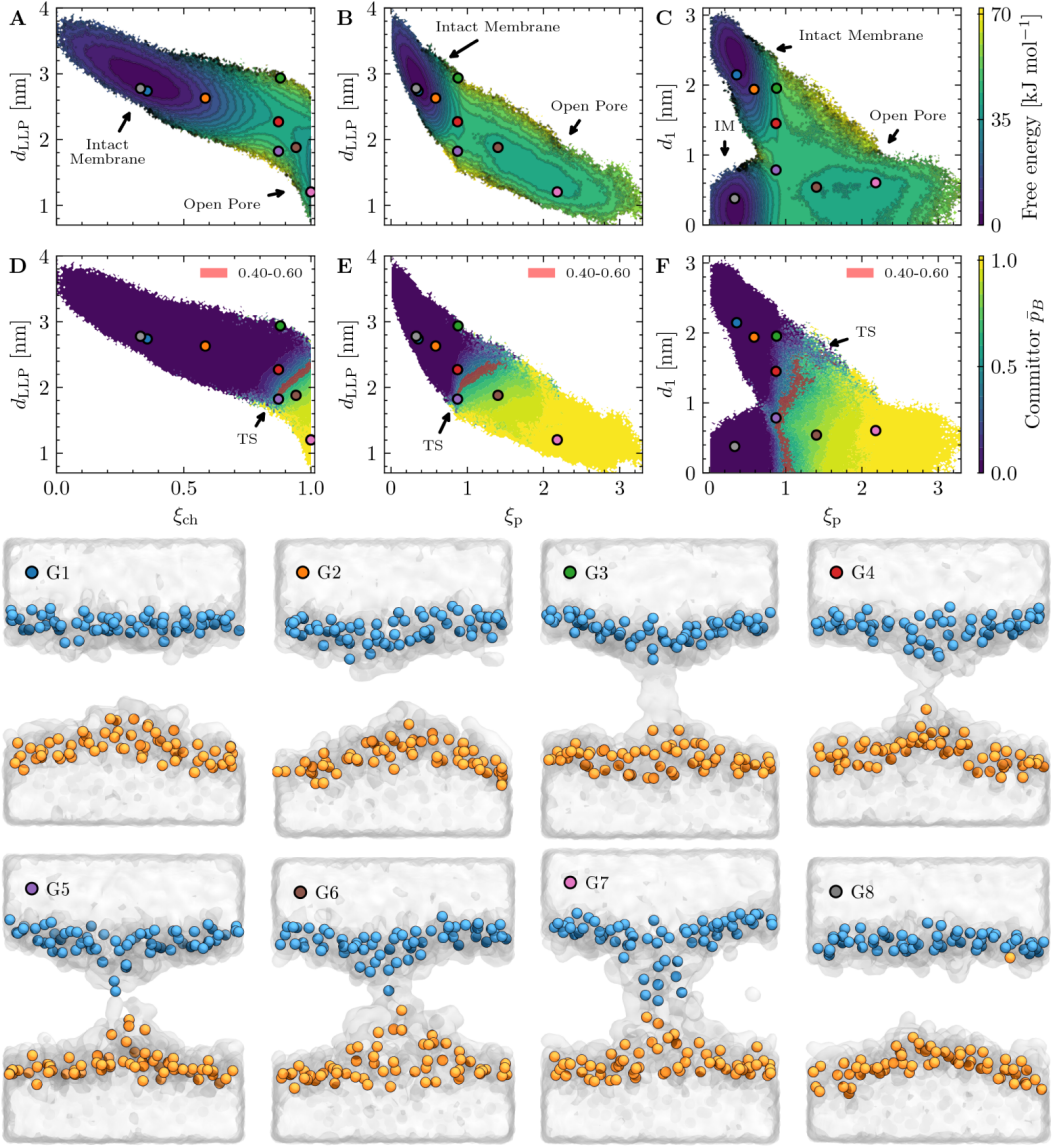
Panels A–F: The relationship between polar defects, membrane thinning, lipid translocation, and toroidal pore formation from defect-free lipid bilayers. Panels A–C show the calculated free energy surfaces, while panels D–F display the corresponding average projected committor functions. TS stands for transition state and IM stands for intact membrane (defect-free membrane). Free energy values exceeding 70 kJ mol^–1^ have been flattened to enhance color contrast. Panels G1–G8: Eight representative geometries with different ξ_ch_, ξ_P_, ξ_LLP_ and *d*_1_ values, centered based on the predicted ξ_ch_ cylinder center. Panels A–G illustrate that configurations with the same ξ_ch_ and ξ_P_ may correspond to different *d*_LLP_ and *d*_1_ values. Lipid phosphorus atoms are shown as spheres, colored blue and orange according to the *d*_1_ grouping scheme. Water molecules are rendered using the “QuickSurf” representation in VMD.^34^ Lipid tails are omitted for clarity.

Using pore-opening and -closing ∞RETIS simulations with stricter definitions of stable states (PF_1_, PF_2_ and PC_1_), the characteristic conditional *P*(λ|λ_*A*_) plateau is observed in Figure 9D–F. By combining pore-formation (PF_1_, PF_2_) with the pore-closing (PC_1_) simulations, the free energy and the averaged committor function, *p̅*_*B*_, can be projected onto arbitrary CVs.^33,35^ Projecting across ξ_ch_ and ξ_p_ in Figure 3B,C, the predicted one-dimensional free energy and transition state appear consistent with the previous reported US results.^10^ Any minor deviations may arise from limited sampling.

The free energy and committor *p̅*_*B*_ for ξ_ch_ and ξ_p_ are also projected against *d*_LLP_, a CV that explicitly considers the proximity of the two leaflets in the region close to the ξ_ch_ cylinder (see Figure 4A,B,D and E). The transition state and open-pore region in the free-energy projections appear better localized with *d*_LLP_, where *d*_LLP_ show that polar defects can emerge without significant lipid translocation into the membrane center. As the committor indicates the probability of a phase point ending up in state B before state A, *p̅*_*B*_ in Figure 4D clearly explains the results of the initial ∞RETIS simulation PF_0_: even for phase points with ξ_ch_ ≈ 0.99, the colored isocommittor surface *p̅*_*B*_ ∈ [0.4, 0.6] indicates that they must also lie in or below *d*_LLP_ < 2.4 nm to have a high probability of reaching the metastable open-pore state. Therefore, rather than describing the transition state by a single CV value ξ_ch_ ≈ 0.88, the isocommittor shows that the transition-state region is better characterized by a linear relationship between ξ_ch_ and *d*_LLP_ in the range of values *p̅*_*B*_ ∈ [0.85, 1.0] and *d*_LLP_ ∈ [1.7, 2.4] nm. Considering the pore expansion projections in Figure 4B,E, ξ_p_ correlates quite linearly with *d*_LLP_ and may together provide a simple physical interpretation for pore formation: the degree of local membrane thinning, reflected by both local leaflet proximity and the size of the polar defect, determines the transition state and the transformation from a defect-free bilayer into a toroidal-shaped open-pore state.

Qualitatively, the free energy and the averaged-committor surfaces are in agreement. However, discrepancies can still be observed, such as the location and width of the transition-state region. As the committor also incorporates dynamical information, its more *narrow* isocommittor surface *p̅*_*B*_ ∈ [0.4, 0.6] provides a better localization of the transition-state region. The difference may indicate that other relevant degrees of freedom remain averaged out, even when including the additional CV *d*_LLP_. We therefore expect more optimal CVs to exist, those that account for both aspects of membrane thinning as well as possible asymmetric and geometric shape effects, such as the toroidal pore transformation process.

We note that simulated bilayers containing 128 lipids (or fewer) are known to suffer from finite size effects, as the open-pore state grow larger in ξ_p_ and becomes more stable with increasing system size in free-energy calculations across ξ_p_.^10,11^ However, the ξ_ch_, ξ_p_ transition-state energy and location appear relatively independent.^10,11^ As the results from the simulated 128-lipid DMPC bilayer are consistent with those from the 256-lipid DMPC and POPC bilayer simulations presented in Section III D, our pore-formation analysis is likely unaffected by these finite-size effects.

### 3.2 Lipid Translocation and Pore Closing

The translocation of lipids between bilayer leaflets was occasionally observed during the sampling of pore-opening events in simulations PF_1_ and PF_2_, consistent with previous studies.^4,36,37^ Given an initial bilayer with a symmetric lipid count of 64–64, we categorized the observed lipid translocation events into two types: (i) translocation events that leave the bilayer composition unchanged, and (ii) translocation events that result in asymmetric bilayer counts, deviating from the original 64–64 configuration. While coupled translocation of two lipids between opposite leaflets (flip and flop) was observed, the most frequently sampled lipid-translocation event involved only a single lipid, resulting in a −1/+1 bilayer state. This is shown in Table 1, which sorts the sampled lipid-translocation trajectories from simulations PF_1_ and PF_2_ according to the change in *d*_CNT_ between their first and last frames, Δ*d*_CNT_. Here, Δ*d*_CNT_ = 0 identifies the “flip and flop” lipid-translocation category, whereas Δ*d*_CNT_ > 0 denotes the number of lipids translocating from one leaflet to the other. For example, while all initial trajectories in simulation PF_2_ were initiated from state *d*_CNT_ = 1, occasional lipid-translocation events promoted the exploration of *d*_CNT_ = 2 as well, as shown by the conditional free-energy contour in Figure 9B.

**Table 1:** Lipid Translocation Data for Simulations PF1, PF2, and PC2^a^

Simulations	CV	0	1	2	3	4	5	PF
PF_1_, PF_2_	Δ*d*_CNT_	7	97	7	1	0	0	716
PC_1_	*d*_CNT_	2128	3057	703	339	38	0	-

a For simulations PF_1_ and PF_2_, the table lists a simple count of all sampled lipid translocation trajectories, where Δ*d*_CNT_ = 0 indicate simultaneous lipid translocations from both leaflets, while Δ*d*_CNT_ > 0 represents asymmetric lipid translocations from a single leaflet. These trajectories can be either reactive (λ_*A*_ → λ_*B*_) or unreactive (λ_*A*_ → λ_*A*_) . The last column lists the total number of sampled pore formation (PF) trajectories for comparison. For simulation PC_1_, the entries indicate the final *d*_CNT_ states reached by the sampled reactive pore-closing trajectories.

The relationship between pore formation and lipid translocation can be visualized by computing the free energy (and committor) as a function of *d*_1_ against ξ_p_, as shown in Figure 4C,F. A strong correlation is observed: successful translocation events require the formation of polar defects spanning the membrane. It should be noted, however, that lipid translocations can also be interpreted as a result of pore closure. Once a pore has opened, the return to a defect-free, symmetric bilayer with a + 0/-0 lipid count may not be guaranteed. Instead, in the pore-closing ∞RETIS simulation PC_1_, the formation of defect-free bilayers with asymmetric leaflet counts ranging between −0/+0 to −4/+4 is observed, as shown by the end *d*_CNT_ state of reactive trajectories in Table 1. Perhaps surprisingly, the majority of the sampled reactive trajectories appear to end up in asymmetric bilayer states with *d*_CNT_ > 0, although the difference between *d*_CNT_ = 0 and *d*_CNT_ = 1 may reflect insufficient sampling.

A natural question arises: does the pore-formation kinetics differ significantly when starting from different defect-free states (*d*_CNT_ = 0, 1, 2, ···) in a 128-lipid bilayer system? Attempts to answer this question motivated the separation of the pore-formation study into two ∞RETIS simulations, PF_1_ and PF_2_. When comparing their “reaction rates” (listed in Table 2), simulations PF_1_ and PF_2_ appear largely indistinguishable. Thus, one can safely ignore the specific contribution of leaflet asymmetry when simulating even larger lipid systems.

**Table 2:** Simulation and Kinetics Results for PF_1_, PF_2_, and PC_1_ ^a^

	PF_1_	PF_2_	PC_1_
Accumulated wall time [days]	26.8	15.4	28.6
Unique accepted paths [μs]	38.2	18.8	29.6
Total time step evaluations [μs]	45.9	23.0	39.7
Sampled accepted paths	42474	23978	37105
Average acceptance ratio [%]	97	96	87
Sampled reactive paths	409	388	6265
Reactive path lengths [ns]	19.0	16.1	1.9
Flux [ns^–1^]	4.12	0.85	5.12
Crossing probability	1.15 × 10^–9^	8.33 × 10^–9^	2.09 × 10^–3^
Rate [ns^–1^]	4.73 × 10^–9^	7.12 × 10^–9^	1.07 × 10^–2^
Rate error [%]	35	60	65
Pore formation [%]	94	93	-
Pore formation rate [ns^–1^]	4.45 × 10^–9^	6.22 × 10^–9^	-

a The second row lists the total accumulated time covered by uniquely sampled reactive paths, which was used for all rate constant and residence time calculations. In contrast to the “simple” path counting shown in Table 1, the averages reported here are computed using proper path weights,^22^ such as the pore formation probability of reactive trajectories in PF_1_ and PF_2_.

Even though considering different *d*_CNT_ states appears inconsequential for our simulated 128-DMPC system, the fact that returning to the original *d*_CNT_ state may not be guaranteed after pore closure raises concerns that free-energy profiles for pore formation could become ill-defined. In particular, without specific consideration of individual lipid-leaflet populations in more complex bilayers that exhibit heterogeneous or asymmetric properties, pore opening may lead to irreversible compositional changes, rendering the original state inaccessible once the pore recloses. For such cases, conditional free-energy profiles, defined purely from trajectory data points originating in λ_*A*_ (and thus not weighted by λ_*B*_ → λ_*B*_/λ_*A*_ trajectories), may offer a more robust framework for understanding the thermodynamics of pore dynamics.

### 3.3 Local Membrane Thinning Determines Pore Formation Sites

As replicas ascend and descend the replica-exchange ladder in the ∞RETIS pore-opening simulations (see Figure 5A), one might expect that individual replicas would randomly form and dissolve polar defects at arbitrary locations across the membrane. This expectation is particularly reasonable given that the simulated bilayer is compositionally homogeneous (composed solely of DMPC lipids) and that the ∞RETIS simulation applies no external biases to localize or stabilize polar defects.

**Figure 5:**
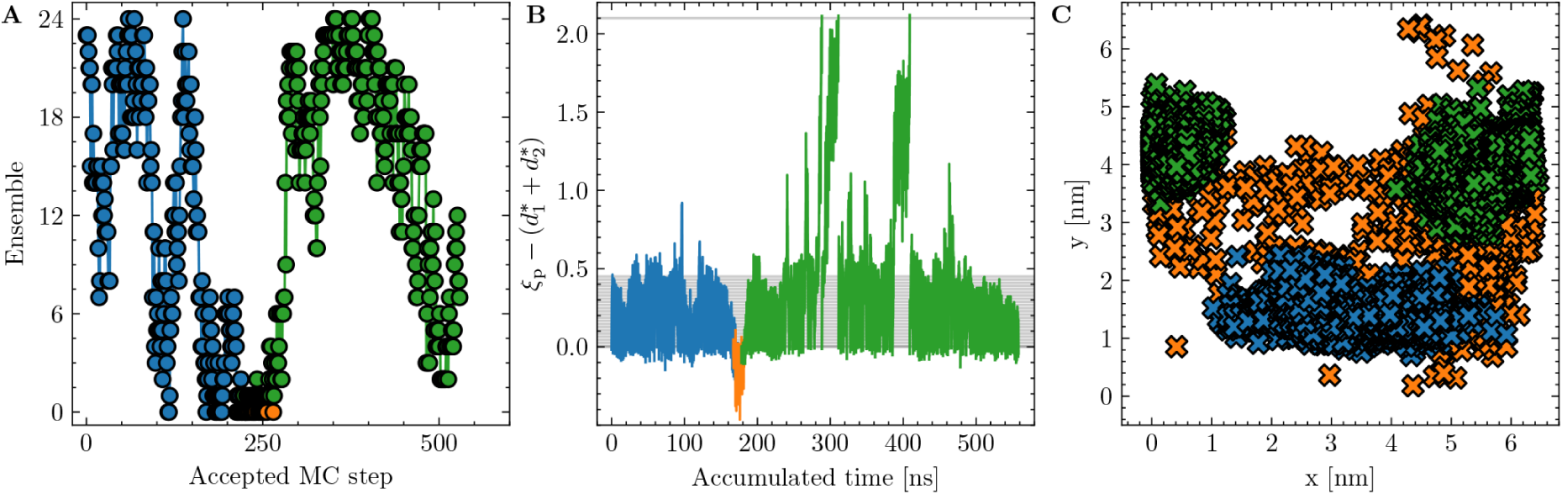
Progression of a replica through the available path ensembles during one of the PF_1_ simulations. The time spent in the [*i* ^+^] ensembles is indicated in blue and green, and in the [0^–^] ensemble in orange. Because a replica is fractionally “swapped” among all available ensembles during each ∞RE move, Panel A shows the ensemble in which the replica resides after each consecutively accepted shooting move. Panel B shows the corresponding order parameter of the replica, defined as $\lambda =\xi_{p}-\left(d_{1}^{*}+d_{2}^{*}\right)$ , plotted against the accumulated simulation time for each uniquely accepted path. Panel C shows the predicted *x* – *y* coordinates of ξ_ch_ cylinder center for all accumulated path points of the replica. As shown, cylinder motion is relatively slow within the [*i* ^+^] ensembles. Significant shifts occur primarily during exchange moves between [0^+^] and [0^–^] ensembles, facilitating broader sampling across the membrane plane (blue → orange → green).

By using the *x* and *y* coordinates of the cylinder defined in ξ_ch_ as a proxy for the center of local pore formation, the position of the polar defect (and local thinning) can be estimated for any MD frame. Interestingly, for paths sampled in the [0^+^] ensembles, the polar defect remains highly localized within a specific *x* – *y* region of the membrane. Neither shooting moves nor replica exchanges, even down to the [0^+^] ensemble, cause significant diffusion of the pore location across the membrane plane (see Figure 5B,C).

This lack of lateral pore diffusion can be attributed to the underlying potential-energy landscape. The sampled [*i* ^+^] paths predominantly explore the barrier region associated with pore opening and closing, limiting the opportunities to visit other regions of phase space before reaching the terminal interfaces λ_*A*_ or λ_*B*_. Notably, the initial and/or final positions of the [*i* ^+^] paths, configurations within λ < λ_*A*_ that should be readily sampled by brute-force MD, also show little deviation in pore location.

This rigidity in the cylinder’s *x* – *y* coordinates suggests that the most probable location for polar-defect formation in defect-free DMPC bilayers can already be inferred near the λ_*A*_ region. Although ξ_ch_ was originally developed to compute the free energy of polar-defect formation across bilayers, the calculated cylinder *x – y* coordinates can also be interpreted as identifying the locally thinnest regions of the membrane, regardless of whether a continuous polar defect is present or not.

Building upon Section III A and III B, we propose a unified mechanism describing the formation of polar defects, metastable pores, and lipid translocation events, as follows:

(1) Natural thermal fluctuations in the bilayer give rise to local regions that are thinner (or thicker) than the membrane average (Figure 1D).
(2) Polar defects are more likely to nucleate in these locally thinned membrane regions, potentially spanning the bilayer even without significant lipid movement toward the membrane center. The transition state is reached only when both sufficient (lipid) thinning and a large enough polar defect coincide (Figure 1E).
(3) In this region, multiple lipids may translocate into the membrane center, stabilizing the defect into a toroidal-shaped metastable open pore (Figure 1F). Movie S1 shows the whole Figure 1D→E→F transition.
(4) Lipid translocation between bilayer leaflets occurs as a result of the closure of localized membrane thinning (Figure 1F→E→D or E→D). Movie S2 shows an example lipid translocation trajectory.

While a replica’s ξ_ch_-cylinder coordinates remain relatively fixed during sampling in the [*i* ^+^] ensembles, much greater mobility is observed when a replica is swapped into the [0^–^] ensemble (which explores λ < λ_*A*_), as shown by the orange scatter points in Figure 5C. Through another point-exchange move between the [0^–^] and [0^+^] ensembles, the replica in Figure 5 explores pore formation, shown by the green data points.

We therefore anticipate substantially more replica “round trips” are required for a single replica to fully explore the *x* – *y* plane of the membrane. Without utilizing replica-exchange moves, however, the sampled pore-formation region would likely remain localized indefinitely or diffuse only very slowly. While the predicted pore-opening kinetics will likely remain similar without replica exchange for our homogeneous lipid-bilayer system, multiple, distinct pore-opening pathways may exist in more complex bilayers. In such systems, replica-exchange moves are therefore essential for exploring orthogonal degrees of freedom and obtaining well-sampled statistics.

### 3.4 *Inf-init*: Likely Reaction Pathways Starting from a Defect-Free Bilayer Configuration

A general prerequisite and challenge for path-sampling algorithms is that an MD trajectory connecting two metastable states must first be obtained before shooting moves can be effectively applied. Due to the rarity of such transitions, methods such as biased or high-temperature simulations have typically been employed to generate the initial path.^37,38^ The external bias is then eliminated through a subsequent series of shooting moves during the path-sampling simulation. So far, path-sampling-based methods generally have not provided an effective way to bypass this demanding initialization and decorrelation process without external assistance. However, leveraging the parallelizability of ∞RETIS, reactive trajectories can now also be efficiently generated directly by starting from equilibrium MD simulations using an ∞RETIS-based algorithm referred to as *Inf-init*. While *Inf-init* has previously been utilized,^30^ it is briefly introduced here in the context of metastable pore formation, with a more detailed description planned for a future publication.

Starting from a defect-free bilayer trajectory obtained from equilibration of the initially constructed configuration (prepared using CHARMM-GUI^25^), *Inf-init* utilizes only configuration points from this equilibrium MD trajectory to initiate a short ∞RETIS simulation. Shooting moves are performed from this pool of configurations, all located near λ_*A*_. These initial shooting moves will likely yield only unreactive paths that both start and end within λ_*A*_. However, if the order parameter λ and the initial interfaces λ_*i*_ are reasonably well chosen, the generated paths are encouraged to climb barriers and explore likely transition pathways toward state λ_*B*_. After a number of shooting moves, the generated path data are used to readjust the interfaces. A new ∞RETIS simulation is then initiated, which typically produces paths that reach even higher maximum λ values. This iterative process continues until reactive trajectories are successfully generated (see the general procedure illustrated in Figures 6 and 7).

**Figure 6:**
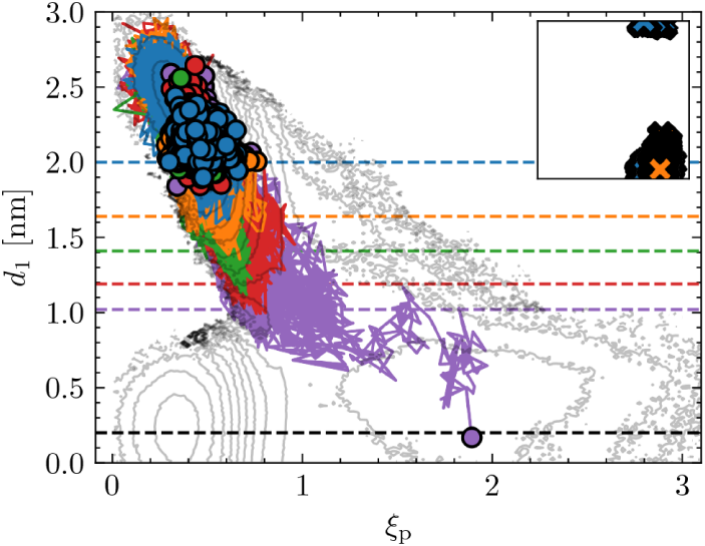
Initialization algorithm *Inf-init* applied to generate a reactive trajectory from a defect-free bilayer configuration using only the CV *d*_1_. The individual sampled paths are shown as line plots, with their end points marked by scatter points. Even without any prior information about polar defects, an open pore is formed as *d*_1_ approaches zero through multiple iterative ∞RETIS simulations (progressing in color: blue → orange → green → red → purple). The colored dashed lines represent the maximum interface value λ_*i*_ for each respective simulation, while the black dashed line indicates λ_*B*_. The complete set of generated *Inf-init* trajectories accumulates to a total of 350 ns of simulation time and aligns well with the projected free-energy contours shown in Figure 4C. The inset scatter plot displays the reactive replica’s cylinder positions, normalized by the box length, across all *Inf-init* rounds.

**Figure 7:**
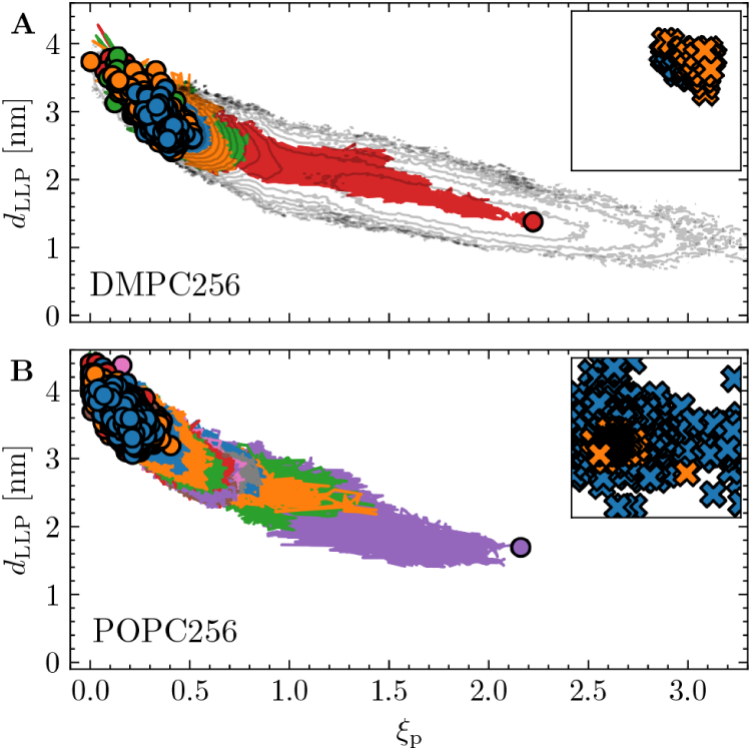
Application of *Inf-init* to generate pore formation trajectories for lipid bilayers consisting of 256 DMPC (panel A) and 256 POPC (panel B) lipids. The *Inf-init* simulations were performed using CV $\lambda =\xi_{p}-\left(d_{1}^{*}+d_{1}^{*}\right)$ , but the trajectories are projected here onto *d*_LLP_ and ξ_p_. Each color corresponds to the set of paths generated within a single *Inf-init* round. For the 256-DMPC system, only four rounds (≈270 ns total simulation time) were required to generate a reactive pore formation trajectory, whereas the 256-POPC system required 13 rounds (≈2000 ns). The free-energy contour shown in panel A is taken from Figure 4B. The inset scatter plots display the cylinder positions of the reactive replica, normalized by the box length, across all *Inf-init* rounds. The final reactive trajectory is shown in orange.

Specifically, although all sampled ∞RETIS paths and the free-energy surface shown in Figure 4C,F indicate that lipid translocation occurs exclusively through pore opening, *Inf-init* is here employed to generate a reactive path in which *d*_1_ decreases from its equilibrium value of approximately 2.5 to 0 nm. In this *Inf-init* simulation, the interfaces are defined solely by λ = *d*_1_ with λ*_A_* = 2.0 and λ*_B_* = 0.2. Without relying on prior knowledge from previous ∞RETIS simulations, the sampled configurations are effectively driven in the ξ_p_ direction as *d*_1_ decreases. Eventually, an open pore forms via traversal of a ≈40 kJ mol^–1^ barrier as *d*_1_ decreases below 0.2, reconfirming the inherent difficulty of lipid translocation in the absence of polar defects, as shown in Figure 6.

A bilayer containing only water and 128 DMPC lipids was utilized for the main ∞RETIS simulations in this work. To assess whether the same general mechanism holds in larger systems, *Inf-init* was also applied to 256-lipid systems containing either DMPC or POPC lipids with ions. As shown in Figure 7, the overall behavior of the unified mechanism described in Section III C remains consistent.

### 3.5 Bilayer Kinetics

For the simulated 128 DMPC system, the PF_1_ results listed in Table 2 predict a pore-formation rate of 4.45 × 10^–9^ events per ns, corresponding to 4.45 pore-formation events per second. Once a pore has formed, its predicted open residence time is only 1/rate ≈ 94 ns. This is significantly shorter than the 400 ns estimated by a recent TPS study for a DMPC bilayer containing 450 lipids, indicating that larger system sizes help stabilize the open-pore state.^37^ However, based on previous studies, the transition state and the overall energy barrier appear to be relatively insensitive to system size beyond 128 lipids. This suggests that the predicted kinetics of pore formation are less affected by finite-size effects than our pore-closing results.^10,11^ We note a recent theoretical study that investigated the relationship between electroporation and pore formation of diphytanoyl phosphatidylcholine (DPhPC) bilayers modeled using CHARMM36.^39^ By combining free-energy calculations with direct observations of pore-formation events at high transmembrane potentials, the authors obtained pore-formation rates consistent with previous experimental measurements. This agreement provides additional confidence in the theoretical predicts reported here.

Given the challenge in calculating kinetic properties in general, the generated ∞RETIS data can also be utilized to estimate the difference between our estimated rate constant *k*_*AB*_ and the rate predicted by more traditional but approximate methods, such as the Transition State Theory (TST) rate constant *k*_TST_. For a predefined reaction coordinate, e.g., ξ_ch_, *k*_TST_ does not account for recrossings of the estimated transition state at $\xi_{\mathrm{ch}}^{‡}\approx 0.88$ . As a result, *k*_TST_ provides only an upper bound on the true rate constant *k*_*AB*_.^15,40^ A corrected *k*_TST_ can be obtained by introducing a transmission coefficient κ, such that *k_AB_* = κ × *k*_TST_. Importantly, ∞RETIS not only yields an estimate of *k*_*AB*_ but also allows *κ* to be computed by postprocessing the trajectory data. Specifically, κ is obtained by counting the fraction of trajectories that end up in the final metastable state after crossing $\xi_{\mathrm{ch}}^{‡}$ (see Figure 8).

**Figure 8:**
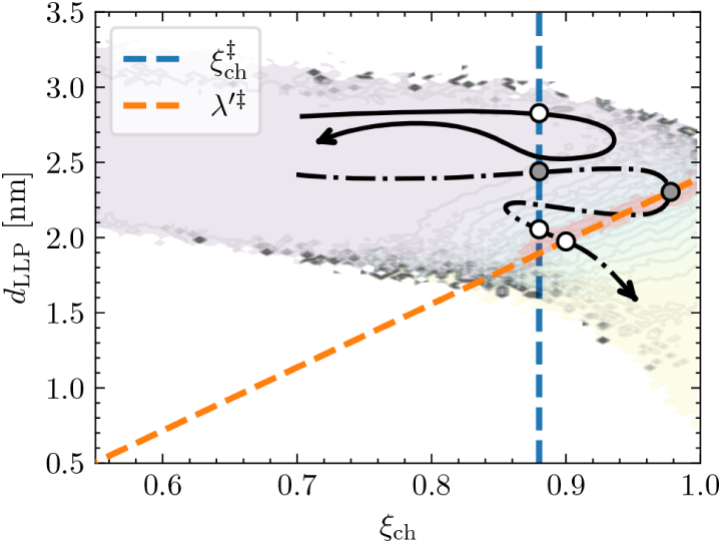
Given the sampled ∞RETIS paths, the transmission coefficient κ for the candidate reaction coordinates $\xi_{\mathrm{ch}}^{‡}$ and λ^′‡^ can be estimated using the effective positive flux counting procedure.^40,41^ For the three crossings of $\xi_{\mathrm{ch}}^{‡}$ with positive momentum, only one (gray) point lies on a reactive path that arrives directly from the reactant basin, yielding *k* = 1/3. For λ^′‡^, only the dotted path is considered, resulting in *k* = 1/2. Paths starting from the open-pore state are also included in the evaluation of κ; however, due to their low statistical weight (*k*_*AB*_/*k*_*BA*_ ≈ 10^–7^, Table 2), their contribution to κ is negligible. The committor contour is taken from Figure 4D.

From conditional path ensemble averages near $\xi_{\mathrm{ch}}^{‡}$ , the transmission coefficient is estimated to *k*_ξ_ = 0.00186 suggesting that *k*_TST_ overestimates *k*_*AB*_ by a factor of ≈500. We note that the saved trajectories consist of data points separated by 1000 timesteps (2 ps), and may thus lead to an undercounting of recrossing events.

If an alternative reaction coordinate is used, λ′ = λ­(ξ_ch_, *d*_LLP_), with the transition state λ^′‡^ defined by the red-highlighted region in Figure 4D, the corresponding transmission coefficient is *k*_λ′_ = 0.00314. This value is approximately 1.6 times higher than that obtained using ξ_ch_ alone, indicating fewer, though still frequent, recrossings through λ^′‡^. This result further suggests that improved CVs may yet to be identified.

### 3.6 ∞RETIS Simulations

Based on Table 2, a total of 409, 388, and 6265 reactive trajectories were sampled for simulations PF_1_, PF_2_ and PC_1_ out of a combined 103,557 trajectories (equivalent to 86.6 μs of data). The total number of time step evaluations performed for each ∞RETIS simulation is reported in Table 2 and includes contributions from the accepted paths, the rejected shooting moves, and the intermediate subtrajectories generated by the wire-fencing move. Except for the higher path ensembles that may generate (and reject) λ_*B*_ – λ_*B*_ trajectories, the acceptance ratio was approximately 100% for most high-acceptance ensembles, as shown in Figure 9P–R. Given the terminal λ_*A*_ and λ_*B*_ interface placements, the average path length for each ensemble ranged from 0.02 to 4 ns, as shown in Figure 9M–O. In comparison, reactive pore formation trajectories required between 10 and 20 ns. While our reported rates appear well converged (see the running estimates in Figure 9G–I), the accuracy may be influenced by finite-size effects. The estimated errors for the flux, conditional crossing probability and the rate are based on a block averaging procedure applied to the running estimate as shown in Figure 9J–L.^42^ The error in the rate is also listed in Table 2. While trajectories ending in specific defect-free *d*_CNT_ states are also counted as “reactive” for simulations PF_1_ and PF_2_, 94% of the reactive trajectories end in a λ_*B*_ state with the last frame having ξ_p_ > 2.0.

**Figure 9:**
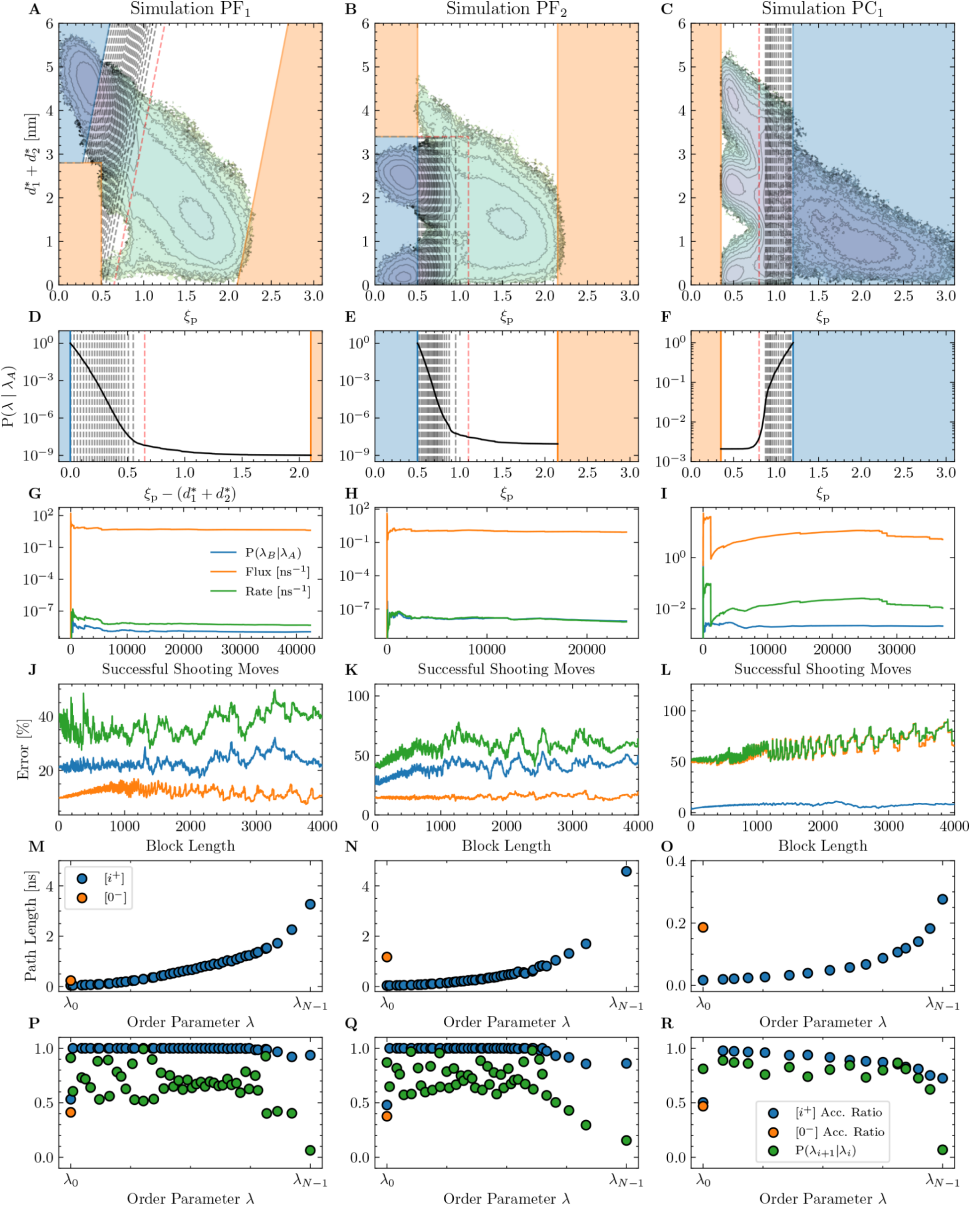
Summary of simulation data for the three ∞RETIS simulations (PF_1_, PF_2_, and PC_1_). Panels A–C show the definitions of the order parameter λ, the stable states A and B (blue and orange), example interface placements, the WF cap interface (dashed red lines), and the conditional free energy. Panels D–F show the simulation crossing probabilities, example interfaces. Panels G–L display the running kinetics and the corresponding error estimates. Panels M–O present the average path lengths sampled in the individual path ensembles, defined by their interface value λ_*i*_. Similarly, panels P–R show the shooting move acceptance ratios and local crossing probabilities for the individual path ensembles.

We end this section with a short discussion on the order parameter utilized for the PF_1_ simulation. As can be qualitatively observed in Figure 9A, the “rectangular orange region” left of ξ_p_ ≤ 0.5 partially overlaps with the computed PF_1_ conditional free-energy contour. Any trajectory starting in λ_*A*_ that enters this region (representing a pore-free bilayer with *d*_CNT_ ≥ 1) is stopped and counted as a sampled reactive λ_*A*_ → λ_*B*_ lipid translocation trajectory by the ∞RETIS program. Due to the inadequate CVs $d_{1}^{*}+d_{2}^{*}$ and *if-else* value definitions, a total of 19 trajectories were improperly labeled as reactive, even if no lipid-translocation events occurred. Thankfully, this number was small, and their exclusion did not significantly affect the analysis. In hindsight, defining a ∞RETIS order parameter λ using ξ_p_ and *d*_CNT_ to separate the *d*_CNT_ states would have simplified the procedure.

## 4 Conclusion

In this work, we characterized the relationship between membrane thinning, polar defects, metastable pores, and lipid translocations in a simulated 128-DMPC bilayer, where the discovery of an additional relevant degree of freedom (local leaflet proximity) resolves the initial discrepancy between US and ∞RETIS results. Although the system studied was relatively simple, consisting solely of water and DMPC lipids, capturing the complexity of pore formation required a broad set of collective variables and detailed visual analyses. Among the mechanistic insights obtained, a particular noteworthy finding is the direct connection that local membrane thinning has on pore formation and lipid translocation. In particular, membrane thinning has been experimentally reported in the context of pore-forming antimicrobial peptides^43,44^ and in the context of lipid translocation through scramblase proteins.^45,46^ These results with our simulations provide a bridge between atomistic observations and macroscopic measurements.

While finite-size effects may influence quantitative aspects of the results, the overall mechanistic pathway from a defect-free bilayer to an open pore state was consistent across larger systems, including 256-DMPC and POPC bilayers. This suggests that the mechanism described here may represent a robust and generalizable framework. Nevertheless, further investigations are needed to assess its validity in more complex settings, such as mixed or asymmetric bilayers, vesicles, or protein–lipid systems. In such environments, rare events may become effectively irreversible, complicating the interpretation of free-energy landscapes.

Finally, this study highlights the power ∞RETIS and *Inf-init*, efficient and highly parallelizable variants of RETIS, as unbiased path-sampling methods for exploring rare events in biological membranes. In particular, these approaches enable the efficient design and flexible implementation of arbitrary CVs without the need for additional force calculations, while allowing postsimulation projection onto alternative coordinates. Taken together, our findings demonstrate both the mechanistic relevance of membrane thinning in pore formation and the broader applicability of ∞RETIS as a robust framework for studying rare events in complex biomolecular systems.

## Supplementary Material





## Notes

Visualization of a sampled ∞RETIS trajectory showing the formation of a metastable pore in a DMPC128 bilayer (MP4)
Visualization of a sampled ∞RETIS trajectory showing a lipid translocation event in a DMPC128 bilayer (MP4)
